# 2,4-Diamino-6-methyl-1,3,5-triazin-1-ium nitrate

**DOI:** 10.1107/S1600536809003900

**Published:** 2009-02-11

**Authors:** Ying Fan, Wei You, Hui-Fen Qian, Jian-Lan Liu, Wei Huang

**Affiliations:** a College of Sciences, Nanjing University of Technology, Nanjing 210009, People’s Republic of China; b State Key Laboratory of Coordination Chemistry, Nanjing National Laboratory of Microstructures, School of Chemistry and Chemical Engineering, Nanjing University, Nanjing, 210093, People’s Republic of China

## Abstract

In the title salt, C_4_H_8_N_5_^+^·NO_3_^−^, a ring N atom of 2,6-diamino-4-methyl­triazine is protonated. Each anion is connected to three neighbouring cations by multiple N—H⋯O hydrogen bonds which, together with N—H⋯N contacts, generate a layer structure.

## Related literature

For 2,6-diamino-4-methyl­triazine compounds, see: Kaczmarek *et al.* (2008 ▶); Perpétuo & Janczak (2007 ▶); Portalone & Colapietro (2007 ▶); Wijaya *et al.* (2004 ▶); Xiao (2008 ▶).
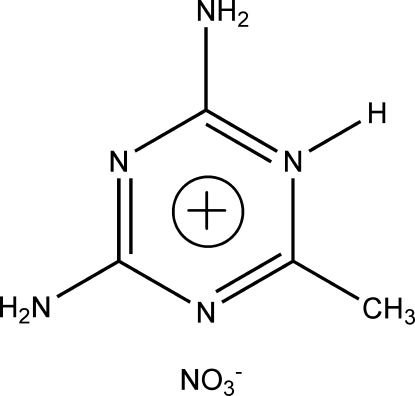


## Experimental

### 

#### Crystal data

C_4_H_8_N_5_^+^·NO_3_^−^
*M* *_r_* = 188.16
Monoclinic, 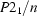

*a* = 7.667 (1) Å
*b* = 10.338 (2) Å
*c* = 9.977 (1) Å
β = 93.384 (2)°
*V* = 789.4 (2) Å^3^
*Z* = 4
Mo *K*α radiation
μ = 0.14 mm^−1^
*T* = 291 (2) K
0.13 × 0.12 × 0.10 mm

#### Data collection

Bruker SMART CCD area-detector diffractometer
Absorption correction: none
4763 measured reflections
1867 independent reflections
1202 reflections with *I* > 2σ(*I*)
*R*_int_ = 0.070

#### Refinement

*R*[*F* ^2^ > 2σ(*F* ^2^)] = 0.046
*wR*(*F* ^2^) = 0.178
*S* = 1.00
1867 reflections
138 parameters
5 restraints
H atoms treated by a mixture of independent and constrained refinement
Δρ_max_ = 0.30 e Å^−3^
Δρ_min_ = −0.38 e Å^−3^

### 

Data collection: *SMART* (Bruker, 2000 ▶); cell refinement: *SAINT* (Bruker, 2000 ▶); data reduction: *SAINT*; program(s) used to solve structure: *SHELXTL* (Sheldrick, 2008 ▶); program(s) used to refine structure: *SHELXTL*; molecular graphics: *SHELXTL*; software used to prepare material for publication: *SHELXTL*.

## Supplementary Material

Crystal structure: contains datablocks global, I. DOI: 10.1107/S1600536809003900/ng2537sup1.cif

Structure factors: contains datablocks I. DOI: 10.1107/S1600536809003900/ng2537Isup2.hkl

Additional supplementary materials:  crystallographic information; 3D view; checkCIF report

## Figures and Tables

**Table 1:** Hydrogen-bond geometry (Å, °)

*D*—H⋯*A*	*D*—H	H⋯*A*	*D*⋯*A*	*D*—H⋯*A*
N2—H2⋯O3	0.88 (2)	2.62 (2)	3.414 (3)	150 (3)
N2—H2⋯O2	0.88 (2)	2.00 (3)	2.831 (3)	156 (3)
N4—H4*D*⋯O1^i^	0.87 (2)	2.17 (2)	3.031 (3)	175 (2)
N4—H4*E*⋯N3^ii^	0.87 (3)	2.24 (3)	3.105 (3)	177 (2)
N5—H5*B*⋯O3	0.90 (1)	2.20 (1)	3.083 (3)	167 (3)
N5—H5*A*⋯O3^iii^	0.89 (1)	2.13 (1)	3.014 (3)	174 (3)
N5—H5*A*⋯O1^iii^	0.89 (1)	2.49 (2)	3.046 (3)	121 (2)

Symmetry codes: (i) 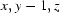; (ii) 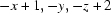; (iii) 
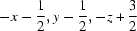
.

**Fig. 1.**
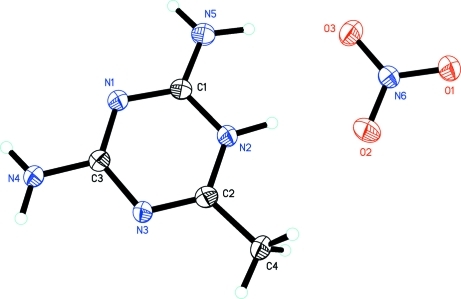
The molecular structure of the title compound with the atom-numbering scheme. Displacement ellipsoids are drawn at the 30% probability level.

**Fig. 2.**
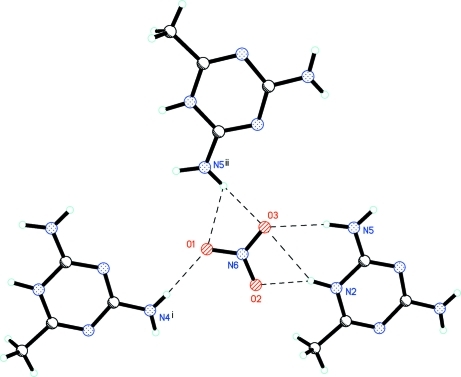
Perspective view of the hydrogen bonding interactions related to every nitrate anion where the hydrogen bonds are shown as dashed lines. [Symmetry codes: (i) x, y-1, z; (ii) -x-1/2, y-1/2, -z+3/2.]

## supplementary crystallographic information

### Comment

The crystal structures of 2,6-diamino-4-methayltriazine with methanol and ethanol solvates (Kaczmarek *et al.*, 2008; Xiao, 2008) and its trifluoroacetate, dimesylamide and hydrogenchlorate (Perpétuo & Janczak 2007; Wijaya *et al.*, 2004; Portalone *et al.*, 2007) have been reported in literature. In this paper, we report the X-ray single-crystal structure of 2,4-diamino-6-methyl-1,3,5-triazin-1-ium nitrate (I).

The molecular structure of (I) is illustrated in Fig. 1. The bond distances and bond angles are similar to those reported structures. All the non-hydrogen atoms of cations and nitrate anions are coplanar with the mean deviation from least-squares plane is 0.0745 (3) Å. The proton is suggested to be delocalized within the aromatic ring although it is added to one of the nitrogen atoms. The molecules of (I) form a layer structure where intermolecular N—H···O, N—H···N hydrogen bonds are found between adjacent molecules (Table 1). Every nitrate is connected with three neighboring cations by multiple N—H···O hydrogen contacts (Fig. 2).

### Experimental

Experimental The title compound was obtained as a by-product from the reaction between CuNO_3_.3H_2_O (180 mg, 1.0 mmol) and 2,6-diamino-4-methayltriazine (935 mg, 5.0 mmol) in methanol (30 ml). Colourless crystals of (I) were obtained by slow evaporation of the mother liquid at room temperature in air after one week. Anal.Calcd. for C_4_H_8_N_6_O_3_: C: 25.55; H: 4.29; N: 44.67%. Found: C: 25.45; H: 4.34; N: 44.56%. Main FT—IR absorptions (KBr, cm^-1^): 3384 (b, s), 2396 (*m*), 1763 (*m*), 1624 (*s*), 1384 (*s*), 825 (*m*), and 456 (*m*).

### Refinement

The methyl H atoms were placed in geometrically idealized positions and refined as riding, with C—H = 0.96 Å and *U*_iso_(H) = 1.5*U*_eq_(C). The H atoms bonded to the N atoms were located in the difference synthesis. Four restraints are used to restrain the bond lengths of N2—H2, N4—H4D, N5—H5A and N5—H5B in order to give similar N—H distances. In addition, one restraint is used to restrain the distance of atoms N1 and H5A so that it is simiar to that between atoms N1 and H4D.

### Crystal data

**Table:**

C_4_H_8_N_5_^+^·NO_3_^−^	*F*(000) = 392
*M**_r_* = 188.16	*D*_x_ = 1.583 Mg m^−^^3^
Monoclinic, *P*2_1_/*n*	Mo *K*α radiation, λ = 0.71073 Å
Hall symbol: -P 2yn	Cell parameters from 1324 reflections
*a* = 7.667 (1) Å	θ = 2.8–26.0°
*b* = 10.338 (2) Å	µ = 0.14 mm^−^^1^
*c* = 9.977 (1) Å	*T* = 291 K
β = 93.384 (2)°	Block, colourless
*V* = 789.4 (2) Å^3^	0.13 × 0.12 × 0.10 mm
*Z* = 4	

### Data collection

**Table:**

Bruker SMART CCD area-detector diffractometer	1202 reflections with *I* > 2σ(*I*)
Radiation source: fine-focus sealed tube	*R*_int_ = 0.070
graphite	θ_max_ = 28.0°, θ_min_ = 2.8°
φ and ω scans	*h* = −8→10
4763 measured reflections	*k* = −12→13
1867 independent reflections	*l* = −12→13

### Refinement

**Table:**

Refinement on *F*^2^	Primary atom site location: structure-invariant direct methods
Least-squares matrix: full	Secondary atom site location: difference Fourier map
*R*[*F*^2^ > 2σ(*F*^2^)] = 0.046	Hydrogen site location: inferred from neighbouring sites
*wR*(*F*^2^) = 0.178	H atoms treated by a mixture of independent and constrained refinement
*S* = 1.00	*w* = 1/[σ^2^(*F*_o_^2^) + (0.106*P*)^2^] where *P* = (*F*_o_^2^ + 2*F*_c_^2^)/3
1867 reflections	(Δ/σ)_max_ = 0.001
138 parameters	Δρ_max_ = 0.30 e Å^−^^3^
5 restraints	Δρ_min_ = −0.38 e Å^−^^3^

### Special details

**Table:**

Experimental. The structure was solved by direct methods (Bruker, 2000) and successive difference Fourier syntheses.
Geometry. All e.s.d.'s (except the e.s.d. in the dihedral angle between two l.s. planes) are estimated using the full covariance matrix. The cell e.s.d.'s are taken into account individually in the estimation of e.s.d.'s in distances, angles and torsion angles; correlations between e.s.d.'s in cell parameters are only used when they are defined by crystal symmetry. An approximate (isotropic) treatment of cell e.s.d.'s is used for estimating e.s.d.'s involving l.s. planes.
Refinement. Refinement of *F*^2^ against ALL reflections. The weighted *R*-factor *wR* and goodness of fit *S* are based on *F*^2^, conventional *R*-factors *R* are based on *F*, with *F* set to zero for negative *F*^2^. The threshold expression of *F*^2^ > σ(*F*^2^) is used only for calculating *R*-factors(gt) *etc*. and is not relevant to the choice of reflections for refinement. *R*-factors based on *F*^2^ are statistically about twice as large as those based on *F*, and *R*- factors based on ALL data will be even larger.

### Fractional atomic coordinates and isotropic or equivalent isotropic displacement parameters (Å^2^)

**Table:**

	*x*	*y*	*z*	*U*_iso_*/*U*_eq_	
C1	0.0367 (3)	0.2000 (2)	0.8411 (2)	0.0350 (5)	
C2	0.3207 (3)	0.2520 (2)	0.9243 (2)	0.0368 (5)	
C3	0.2268 (3)	0.0451 (2)	0.9114 (2)	0.0340 (5)	
C4	0.4496 (3)	0.3560 (2)	0.9556 (3)	0.0488 (6)	
H4A	0.5589	0.3183	0.9872	0.073*	
H4B	0.4074	0.4113	1.0239	0.073*	
H4C	0.4661	0.4057	0.8761	0.073*	
H2	0.129 (4)	0.372 (2)	0.858 (3)	0.067 (9)*	
H4D	0.189 (3)	−0.137 (2)	0.909 (2)	0.034 (6)*	
H4E	0.371 (4)	−0.096 (2)	0.965 (3)	0.044 (7)*	
N1	0.0678 (2)	0.07622 (17)	0.85890 (19)	0.0366 (5)	
N2	0.1626 (2)	0.29135 (19)	0.87137 (19)	0.0373 (5)	
N3	0.3580 (2)	0.13121 (17)	0.94713 (19)	0.0358 (5)	
N4	0.2668 (3)	−0.07814 (18)	0.9311 (2)	0.0415 (5)	
N5	−0.1161 (3)	0.2428 (2)	0.7917 (2)	0.0484 (6)	
H5A	−0.193 (3)	0.1810 (18)	0.771 (3)	0.082 (9)*	
H5B	−0.131 (4)	0.3288 (11)	0.783 (3)	0.066 (9)*	
N6	0.0038 (2)	0.61218 (18)	0.84158 (19)	0.0391 (5)	
O1	−0.0186 (2)	0.73101 (16)	0.84536 (19)	0.0515 (5)	
O2	0.1471 (2)	0.56484 (18)	0.8772 (2)	0.0582 (6)	
O3	−0.1190 (3)	0.54094 (17)	0.7991 (2)	0.0596 (6)	

### Atomic displacement parameters (Å^2^)

**Table:**

	*U*^11^	*U*^22^	*U*^33^	*U*^12^	*U*^13^	*U*^23^
C1	0.0308 (11)	0.0347 (12)	0.0391 (11)	0.0002 (8)	−0.0019 (8)	0.0011 (9)
C2	0.0326 (12)	0.0345 (12)	0.0426 (12)	−0.0011 (9)	−0.0030 (9)	−0.0022 (9)
C3	0.0316 (11)	0.0309 (11)	0.0394 (11)	−0.0013 (8)	0.0010 (9)	−0.0007 (8)
C4	0.0425 (14)	0.0299 (12)	0.0722 (16)	−0.0060 (9)	−0.0120 (12)	−0.0034 (11)
N1	0.0307 (10)	0.0306 (10)	0.0478 (11)	−0.0016 (7)	−0.0035 (8)	0.0007 (8)
N2	0.0337 (10)	0.0280 (10)	0.0493 (11)	0.0009 (7)	−0.0056 (8)	0.0022 (8)
N3	0.0315 (10)	0.0263 (9)	0.0488 (11)	−0.0008 (7)	−0.0054 (8)	−0.0018 (7)
N4	0.0322 (11)	0.0284 (11)	0.0629 (13)	−0.0031 (8)	−0.0070 (9)	0.0015 (8)
N5	0.0351 (11)	0.0422 (13)	0.0661 (13)	0.0015 (9)	−0.0122 (9)	0.0049 (10)
N6	0.0357 (11)	0.0339 (10)	0.0474 (11)	0.0038 (8)	−0.0007 (8)	0.0017 (8)
O1	0.0459 (10)	0.0359 (10)	0.0719 (12)	0.0047 (7)	−0.0045 (8)	−0.0026 (8)
O2	0.0414 (11)	0.0514 (12)	0.0796 (14)	0.0133 (8)	−0.0134 (9)	0.0030 (9)
O3	0.0468 (11)	0.0408 (10)	0.0896 (14)	−0.0073 (8)	−0.0107 (10)	−0.0001 (9)

### Geometric parameters (Å, °)

**Table:**

C1—N1	1.311 (3)	C4—H4B	0.9600
C1—N5	1.321 (3)	C4—H4C	0.9600
C1—N2	1.371 (3)	N2—H2	0.88 (2)
C2—N3	1.298 (3)	N4—H4D	0.87 (2)
C2—N2	1.356 (3)	N4—H4E	0.87 (3)
C2—C4	1.481 (3)	N5—H5A	0.888 (10)
C3—N4	1.322 (3)	N5—H5B	0.900 (10)
C3—N1	1.337 (3)	N6—O2	1.236 (2)
C3—N3	1.375 (3)	N6—O1	1.241 (2)
C4—H4A	0.9600	N6—O3	1.249 (3)
			
N1—C1—N5	121.8 (2)	C1—N1—C3	116.22 (18)
N1—C1—N2	121.5 (2)	C2—N2—C1	118.7 (2)
N5—C1—N2	116.6 (2)	C2—N2—H2	126 (2)
N3—C2—N2	122.7 (2)	C1—N2—H2	115 (2)
N3—C2—C4	121.6 (2)	C2—N3—C3	115.26 (18)
N2—C2—C4	115.7 (2)	C3—N4—H4D	119.0 (17)
N4—C3—N1	119.2 (2)	C3—N4—H4E	117.7 (17)
N4—C3—N3	115.2 (2)	H4D—N4—H4E	123 (2)
N1—C3—N3	125.6 (2)	C1—N5—H5A	114.3 (16)
C2—C4—H4A	109.5	C1—N5—H5B	118 (2)
C2—C4—H4B	109.5	H5A—N5—H5B	128 (3)
H4A—C4—H4B	109.5	O2—N6—O1	120.34 (19)
C2—C4—H4C	109.5	O2—N6—O3	120.2 (2)
H4A—C4—H4C	109.5	O1—N6—O3	119.42 (18)
H4B—C4—H4C	109.5		

### Hydrogen-bond geometry (Å, °)

**Table:**

*D*—H···*A*	*D*—H	H···*A*	*D*···*A*	*D*—H···*A*
N2—H2···O3	0.88 (2)	2.62 (2)	3.414 (3)	150 (3)
N2—H2···O2	0.88 (2)	2.00 (3)	2.831 (3)	156 (3)
N4—H4D···O1^i^	0.87 (2)	2.17 (2)	3.031 (3)	175 (2)
N4—H4E···N3^ii^	0.87 (3)	2.24 (3)	3.105 (3)	177 (2)
N5—H5B···O3	0.90 (1)	2.20 (1)	3.083 (3)	167 (3)
N5—H5A···O3^iii^	0.89 (1)	2.13 (1)	3.014 (3)	174 (3)
N5—H5A···O1^iii^	0.89 (1)	2.49 (2)	3.046 (3)	121 (2)

Symmetry codes: (i) *x*, *y*−1, *z*; (ii) −*x*+1, −*y*, −*z*+2; (iii) −*x*−1/2, *y*−1/2, −*z*+3/2.
